# Lateral compression type 1 fracture fixation in the elderly (L1FE): study protocol for a randomised controlled trial (with internal pilot) comparing the effects of INFIX surgery and non-surgical management for treating patients with lateral compression type 1 (LC-1) fragility fractures

**DOI:** 10.1186/s13063-022-07063-5

**Published:** 2023-02-02

**Authors:** Elizabeth Cook, Joanne Laycock, Mehool Acharya, Michael Ross Backhouse, Belen Corbacho, Laura Doherty, Daren Forward, Catherine Hewitt, Catherine Hilton, Peter Hull, Jamila Kassam, Camila Maturana, Catriona Mcdaid, Jenny Roche, Dhanupriya Sivapathasuntharam, David Torgerson, Peter Bates

**Affiliations:** 1 York Trials Unit, Department of Health Sciences, University of York, Heslington, YO10 5DD UK; 2 Pelvic and Acetabular Reconstruction Unit, Southmead Hospital, Bristol, BS10 5NB UK; 3 Warwick Clinical Trials Unit, Warwick Medical School, University of Warwick, Coventry, CV4 7AL UK; 4 Nottingham University Hospitals, Derby Road, Nottingham, NG7 2UH UK; 5 Cambridge University Hospitals NHS Foundation Trust, Hills Road, Cambridge, CB2 0QQ UK; 6 Bart’s Health NHS Trust, The Royal London Hospital, Whitechapel Road, Whitechapel, London, E1 1BB UK

**Keywords:** INFIX surgery, Lateral compression type 1, LC-1, Pelvic fracture fixation, Elderly patients, Older adults, Fragility fracture, Osteoporotic bone, Pubic ramus fracture, Immobility-related complications

## Abstract

**Background:**

Lateral compression type1 (LC-1) fragility fractures are a common, painful injury in older adults resulting in reduced mobility. The incidence of these fractures is increasing with the growing older adult population. The current standard of care is non-surgical management; however, patients with this injury are at risk of long-term immobility and related complications. INFIX is a pelvic fixation device used in younger patients with high-energy fractures. The device is fitted via a percutaneous technique with no external pin sites and has good purchase even in osteoporotic bone. It therefore has the potential to be well tolerated in patients with LC-1 fragility fractures. INFIX could improve patients’ ability to mobilise and reduce the risk of immobility-related complications. However, there is a risk of complications related to surgery, and robust evidence is required on patient outcomes. This study will investigate the clinical and cost-effectiveness of surgical fixation with INFIX compared to non-surgical management of LC-1 fragility fractures in older adults.

**Methods:**

A multi-centre randomised controlled trial of 600 patients allocated 1:1 to non-surgical management or INFIX surgery. The study will have a 12-month internal pilot to assess recruitment and trial feasibility. The primary outcome will be the patient quality of life over 6 months, measured by the patient-reported EQ-5D-5L. The secondary outcomes will include physical function, mental health, pain, delirium, imaging assessment, resource use, and complications.

**Discussion:**

The L1FE study aims to compare the clinical and cost-effectiveness of surgical and non-surgical management of people aged 60 years and older with LC-1 fragility fractures. The trial is sufficiently powered and rigorously designed to inform future clinical and patient decision-making and allocation of NHS resources.

**Trial registration:**

International Standard Randomised Controlled Trial Number Registry ISRCTN16478561. Registered on 8 April 2019

**Supplementary Information:**

The online version contains supplementary material available at 10.1186/s13063-022-07063-5.

## Administrative information

**Table:** 

Title	Lateral compression type 1 fracture fixation in the elderly (L1FE): study protocol for a randomised controlled trial (with internal pilot) comparing the effects of INFIX surgery and non-surgical management for treating patients with lateral compression type 1 (LC-1) fragility fractures
Trial registration	Trial Identifier: ISRCTN16478561 Registry Name: International Standard Randomised Controlled Trial Number Registry Registered: 8th April 2019 https://www.isrctn.com/ISRCTN16478561?q=ISRCTN16478561&filters=&sort=&offset=1&totalResults=1&page=1&pageSize=10&searchType=basic-search
Protocol version	Protocol V3.2 22nd December 2020
Funding	The National Institute for Health Research Health Technology Assessment programme (reference number: 16/167/57)
Author details	E Cook* J Laycock* M Acharya*** M R Backhouse* & ****** B Corbacho* L Doherty* D Forward**** C Hewitt* C Hilton** P Hull***** J Kassam** C Maturana* C Mcdaid* J Roche* D Sivapathasuntharam** D Torgerson* P Bates** *York Trials Unit, Department of Health Sciences, University of York, Heslington, YO10 5DD **Bart’s Health NHS Trust, The Royal London Hospital, Whitechapel Road, Whitechapel, London, E1 1BB ***Pelvic and Acetabular Reconstruction Unit. Southmead Hospital Bristol. BS10 5NB **** Nottingham University Hospitals, Derby Road, Nottingham NG7 2UH ***** Cambridge University Hospitals NHS Foundation Trust, Hills Road, Cambridge, CB2 0QQ ******current affiliation Warwick Clinical Trials Unit, Warwick Medical School, University of Warwick, CV4 7AL, (work undertaken at *).
Name and contact information for the trial sponsor	Sponsor: Bart’s Health NHS Trust Contact: Dr Mays Jawad, Research & Development Governance Operations Manager, Joint Research Management Office, Queen Mary Innovation Centre, Lower Ground Floor, 5 Walden Street, London, E1 2EF. Telephone: 02078827275. Email: research.governance@qmul.ac.uk
Role of sponsor	The sponsor played no part in the study design and will play no part in the collection, management, analysis, and interpretation of the data; writing of the report; and the decision to submit the report for publication.

## Background

### Research question

What is the clinical and cost-effectiveness of surgical fixation with INFIX compared to non-surgical management of lateral compression type 1 (LC-1) fragility fractures in older adults?

### What are LC-1 fractures and how prevalent are they?

LC-1 fractures are a common fragility fracture in older adults, especially those with osteoporosis. They typically involve a fracture of the pubic ramus, which is perceived by the patient as groin pain when they mobilise. There is usually also a ‘buckle’ fracture to the sacrum posteriorly, which is felt as low back/buttock pain when moving the legs. LC-1 fragility fractures result from a low-energy fall from a standing height or less and most often affect women, with the likelihood of fracture increasing with age [1–3].

LC-1 fractures are often painful, with pain made worse by movement, which inevitably results in a period of reduced mobility. While this period may only last for a month or two, it is estimated that 25% of patients experience pain for up to 5 years afterwards [4]. Patients with LC-1 fractures usually fall into two main groups: those that can mobilise, albeit with some degree of pain, and those where pain strongly affects a patient’s ability to ‘get going’. Patients that fail to mobilise due to ongoing pain are at greater risk of immobility-related complications [5]. These complications include respiratory tract infections, urinary tract infections, pressure sores, and venous thromboembolic events (VTE) such as deep vein thrombosis or pulmonary embolism [5, 6]. These individuals are also at risk of systemic sarcopenia (irreversible muscle wasting), disabling loss of confidence, and permanently decreased levels of independence, often leading to increased care requirements. Inability to return to independent living can result in utilisation of intermediate care or residential facilities [7, 8]. Such as the loss of confidence and muscle strength/conditioning in certain patients following LC-1 fracture, they do not regain their pre-injury level of ambulation or their prior independence with activities of daily living [1, 3, 9, 10]. Additionally, individuals with LC-1 fractures have reported emotional stress, family strain, employment and financial difficulty, sleep disturbance, and anxiety [11]. Pelvic fractures are also associated with increased mortality, with a total in-patient mortality rate of 9%, and an all-cause mortality rate within 3 months of fracture of 13% [12]. All-cause mortality following pelvic fracture is around 50% at 3 years [2]. Progress in the treatment of LC-1 fractures is needed to improve outcomes and quality of life (QOL).

With an ageing population, the incidence of pelvic fractures is rising. The UK age-specific incidence of pelvic fractures (based on a single centre) has increased from 39.6/100,000 (95% CI: 31.8 to 48.1) in 1997 to 71.6/100,000 (58.4 to 81.0) in 2007–2008 amongst people 65 years and older; 84% of these had pubic rami fractures [13]. This increase is supported by evidence from other countries, e.g. in Finland (based on national data) where the incidence, amongst people 60–years and older, has increased from 20/100,000 in 1970 to 92/100,000 in 1997 [14]. The estimated median treatment cost of pelvic ring fractures in Europe (acute hospital, surgery, rehabilitation, physiotherapy, and work-related absence) is €33,710 per patient (interquartile range €23,266 to €51,012), which is more costly than hip fractures [15].

### The current standard care for LC-1 fragility fractures

The current standard treatment for LC-1 fragility fractures in the UK is non-surgical management and to ‘mobilise as pain allows’ [5, 16, 17]. For many patients, this is successful, and they are able to get up within a few days of injury and mobilise with an assistive device. However, pain can lead to immobility, leaving this predominantly older adult population at risk of significant complications.

Unlike LC-1 fragility fractures, fractures involving the upper end of the femur in older adults (also known as ‘hip fractures’) are invariably treated surgically, with either internal fixation of the bone or joint replacement being mandated within 36 h of injury [18, 19]. This is because patients with conservatively managed hip fractures are known to heal significantly worse than those that undergo surgery, and the long-term risks to the patient resulting from prolonged immobility due to pain are much more severe than the immediate risks of surgery. Despite LC-1 fractures being similarly disabling for some patients in terms of pain and immobility and occurring in the same patient group as hip fractures, to date, it has not been shown whether or not older adult patients with LC-1 fractures would heal significantly better with surgery than conservative management. Traditional pelvic implants carry poor ‘bite’ or ‘purchase’ in the low-quality osteoporotic bone around the pelvis, and surgeons have been reluctant to offer surgery to patients with LC-1 fractures. The current standard of care for LC-1 fractures is for patients to be prescribed pain relief medication and mobilise with physiotherapist input as best they can until the fracture eventually heals.

Until recently, there has not been an effective operation to treat osteoporotic LC-1 fractures. External fixators, consisting of pins inside the pelvis connected to bars and clamps outside of the skin, are cumbersome, poorly tolerated, and carry a high incidence of pin site infections and soft tissue problems [20]. An alternative is the surgical fixation of the back of the pelvis with ilio-sacral screws [3]. Although these are effective for certain fracture configurations, in the majority of older patients, these screws carry poor ‘purchase’ in osteoporotic bone, leading to ineffective fracture stabilisation and persistence of pain [5].

### Surgical fixation with INFIX device

The INFIX is an anterior pelvic fixation device that resembles a traditional external fixator, in that it has screws that are secured into the pelvic bone, and these are connected by a metal bar across the front of the patient. Unlike traditional external fixation devices, INFIX is fitted internally, sitting entirely underneath the patient’s skin, with no external metalwork visible. This has two potential benefits over external fixation: it is less cumbersome and inconvenient to patients, compared with pins, clamps, and bars protruding out of the skin. It also does not have pin sites (where the bone pins exit through the skin), which make traditional external fixation very susceptible to local infection. The INFIX technique involves the percutaneous placement of screws in the pelvic bone and connects them with a bar under the skin [21]. The pelvic bone where the screws are placed is generally strong and easy to visualise intra-operatively, even in very osteoporotic bone, making internal fixation (e.g. INFIX) a much more appealing surgical option for these fractures. Although a proportion of implants need to be removed; this is usually done as a day-case procedure. INFIX is widely used in younger patients with high-energy fractures. It is now a well-described technique with a number of peer-reviewed series confirming its safety [22]. It is therefore a widely practised, rather than ‘novel’ technique and is technically straightforward to carry out.

### Justification for the trial

A systematic review found no robust evaluations, particularly randomised controlled trials (RCTs), of the effectiveness of internal fixation with INFIX in patients with osteoporotic LC-1 fractures [23]. The review identified five case series, with four being retrospective. Participants were 64 or over, and most had sustained their injury from a low-energy fall. A variety of fixation techniques were used. Of the 225 patients in the five studies, most had internal devices, with 25 having external fixation; most patients had more than one type of fixation.

In the single series evaluating INFIX alone, 19 of the 29 patients had LC-1 fractures [24]. Six patients had an anterior fixation with INFIX alone, and the remaining 23 had INFIX with additional internal fixation. Post-operatively, 22 of the 29 (76%) returned to their premorbid walking status, and a further six patients had some deterioration but remained ambulatory. Chronic pain (*n* = 3, 10.3%) and painful lateral femoral cutaneous nerve hyperaesthesia (*n* = 8, 27.5%) were prevalent after INFIX fixation. Other complications reported included failure to return to premorbid walking status, infections, implant loosening, pneumonia, and thrombosis.

Our search of ClinicalTrials.gov for ongoing studies identified a trial in the USA of surgical versus non-surgical management of patients aged between 18 and 80 with lateral compression type 1, 2, and 3 pelvic fractures in 130 participants. The aim of this trial is to determine which patients would benefit from early surgical stabilisation [25]. We are also aware that NIHR Research for Patient Benefit (RfPB) has funded TULIP, a feasibility trial of surgical versus non-surgical treatment of LC-1 fractures of the pelvis in non-fragility fracture patients. This study is complementary to TULIP as it investigates their excluded population (i.e. fragility fracture patients).

The pelvic fracture community is at a key point in considering adopting internal fixation devices such as INFIX in the management of LC-1 fractures. In August 2016, we conducted a survey of 32 pelvic surgeons across the UK, of whom 29 responded; 70% felt there was a potential role for treating older patients with low-energy LC-1 fractures with INFIX if they fail to mobilise effectively due to pain.

We now have a device which has the potential ability to effectively stabilise LC-1 fractures in older adults, thereby potentially allowing them to mobilise sooner and prevent long-term complications of immobility. The intervention is increasingly used by pelvic surgeons in major trauma centres (MTCs) for people with high-energy fractures. However, more evidence of effectiveness is needed to evaluate the use of the INFIX device in older patients with fragility fractures. We will investigate the effectiveness, safety, and cost-effectiveness of internal fixation with devices such as INFIX compared to non-surgical treatment in older adults.

We are aware that this trial may be challenging to recruit as the intervention involves an additional surgery not performed in standard care; furthermore, the target population (older adults) is a patient group that may have reservations about having surgery. A Patient and Public Involvement (PPI) group has had input into the recruitment and consent process and helped to make our patient information sheets accessible. To test the feasibility of recruiting to this study, an internal pilot phase will be included.

### Objectives

The objectives of this trial are to:

1. Undertake a 12-month internal pilot to obtain robust estimates of recruitment and confirm trial feasibility.
2. Undertake a parallel group multi-centre RCT to assess the effectiveness of surgical fixation with INFIX versus non-surgical management of LC-1 fragility fractures in older adults. The primary outcome is the average patient quality of life and function, over 6 months, assessed by the patient-reported EuroQol 5 Dimension, 5-Level Scale (EQ-5D-5L) measured at baseline, 2 weeks, 6 weeks, 12 weeks, and 6 months.
3. Undertake an economic evaluation to compare the cost-effectiveness of surgical fixation compared to non-surgical management, to determine the most efficient provision of future care, and to describe the resource impact on the NHS for the two treatment options.
4. Undertake a long-term review of patient wellbeing (EQ-5D-5L and mortality) 12 months after entering the trial.

## Methods

### Trial design

This study is a multi-centre, randomised controlled, parallel-group superiority trial, with a 12-month internal pilot phase to assess the assumptions about recruitment and provide guidance on optimising the trial processes before proceeding to the main trial phase. The allocation ratio of non-surgical management to INFIX surgery is 1:1.

### Study setting

The study will be undertaken at up to 21 NHS MTCs across England, Scotland, Wales, and Northern Ireland; planned sites are shown in Table 1. All sites will have surgeons who are experienced in doing these operations or who have the capacity to be trained.

**Table 1:** Participating NHS Trusts

NHS Trust	
Bart’s Health NHS Trust	
North Bristol NHS Trust	
Cambridge University Hospital NHS Foundation Trust	
Kings College Hospital NHS Foundation Trust	
Brighton and Sussex University Hospitals NHS Trust	
Oxford University Hospitals NHS Foundation Trust	
South Tees Hospitals NHS Foundation Trust	
Cardiff and Vale University LHB	
University Hospitals Coventry and Warwickshire NHS Trust	
St George’s University Hospitals NHS Foundation Trust	
Imperial College Healthcare NHS Trust	
Liverpool University Hospitals NHS Foundation Trust	
Northern Care Alliance NHS Foundation Trust	
NHS Grampian, Aberdeen Royal Infirmary	
University Hospitals Plymouth NHS Trust	
NHS Lothian	
Sheffield Teaching Hospitals NHS Foundation Trust	
Hull and East Yorkshire Hospitals NHS Trust	
Leeds Teaching Hospitals NHS Trust	
University Hospital Southampton NHS Foundation Trust	
NHS Greater Glasgow and Clyde	
Nottingham University Hospitals NHS Trust	
University Hospital Birmingham NHS Foundation Trust	
University Hospitals of North Midlands NHS Trust	
Portsmouth Hospitals NHS Trust	

### Eligibility criteria

Patients who meet all the inclusion criteria and none of the exclusion criteria will be eligible for the trial. Eligibility will be assessed by research nurses/associates and must be confirmed by a surgeon or clinician authorised in the trial delegation log prior to recruitment.

The following are the inclusion criteria:

- Patients aged 60 years or older.
- An LC-1 pelvic fracture, arising from a low-energy fall from standing height or less.
- Patient unable to mobilise independently to a distance of around 3 m and back due to pelvic pain (or perceived pelvic pain) 72 h after injury. Use of a walking aid and verbal guidance are permitted; however, physical assistance is not.

The following are the exclusion criteria:

- Unable to perform surgery within 10 days of injury.
- Surgery is contra-indicated due to soft tissue concerns or because the patient is not fit for anaesthetic (spinal or general).
- Patients who were non-ambulatory or required physical assistance to walk, prior to their injury (use of walking aid is permitted).
- Concomitant injury or poly-trauma that impedes mobilisation.
- Fracture configurations not amenable to internal fixation using INFIX, with or without ilio-sacral screws.
- Patients who test positive for COVID-19 within 72 h of admission (applicable only where testing is standard of care).

Participating surgeons must be familiar with the surgical procedure (have previously conducted 10 or more INFIX procedures or undergo training until the CI confirms that they are sufficiently experienced). Level of experience will be recorded, and no grade of the surgeon will be excluded from performing the procedure. In addition, all surgeons will be required to watch a training video and read a summary guidance document.

There will be no specific requirements in place on who can deliver the non-surgical rehabilitation which will be delivered in line with routine practice at the participating site.

Key trial outcomes are patient-reported and not validated in languages other than English. Patients who do not have adequate verbal or written English skills or do not have family or friends who can sufficiently support them in the completion of the questionnaires will not be recruited.

### Informed consent

Once eligibility is confirmed, hospital research staff will obtain written informed consent from patients who have the capacity. This study will also include patients who lack capacity, and in this instance, consultee agreement or consent will be obtained in line with national guidelines.

Routine capacity assessments performed by the clinical staff on admission will be used in conjunction with the research staff’s judgement to determine whether the patient has the capacity to provide consent.

Consent or consultee agreement will be sought for follow-up beyond the duration of the trial to allow the possibility of future long-term follow-up including the use of routinely collected Hospital Episode Statistics (HES) and Office of National Statistics (ONS) data.

### Interventions

#### Non-surgical management

This is the standard care for LC-1 fragility fractures in this patient population in the UK. Patients are routinely administered pain relief and seen by a physiotherapy team who mobilises patients as pain allows.

#### INFIX surgery

INFIX is a type of anterior internal fixation device; it is fitted internally underneath the patient’s skin. The technique involves percutaneous placement of long pedicle screws within the pelvic bone, these are connected by a metal rod across the front of the patient under the skin. As this is a pragmatic study, surgeons can use their preferred INFIX device. The primary fixation for every patient is INFIX. If the surgeon feels that the fracture configuration in a patient warrants supplementary ilio-sacral screw fixation, this is permissible under the trial, provided adequate intra-operative pelvic imaging can be achieved. Within this study, INFIX surgery is required to be performed within 10 days of injury.

All participants will receive pain relief and physiotherapy as per standard care at the participating site; they will also be provided with a trial rehabilitation leaflet. This leaflet details suggested exercises to perform and is intended to supplement and not replace advice given by the site physiotherapy team. Instructions will state ‘immediate weight bearing, as pain allows’. For both groups, the goals of physiotherapy are to improve function, strength, and range of movement in both legs, while aiming to get patients back to independent mobility as soon as possible.

If a patient randomised to the surgical arm tests positive for COVID-19 prior to their surgery, they will cross over to the non-surgical arm. If a patient randomised to INFIX surgery later requests not to have surgery, then non-operative management should be given. INFIX surgery is not routinely offered as standard care; therefore, if a patient randomised to non-surgical management requests INFIX surgery, the site may be unable to offer this.

In either the non-operative management or the INFIX surgery group, if any patient’s course is complicated by excessive pain when mobilising, a repeat radiograph is clinically indicated, followed by a review by a pelvic surgeon, as would be the normal standard of care. No concomitant care is prohibited, data will be collected on all clinic visits and medication required during the trial. This includes monitoring the number and duration of physiotherapy sessions as well as the pain relief medication that patients in both groups receive as part of their rehabilitation.

There are no special compensation arrangements for this study; the normal National Health Service complaints procedure is available to anyone who has concerns. This study will be sponsored by Bart’s Health NHS Trust. NHS indemnity scheme will apply.

### Outcomes

#### Primary outcome: health-related quality of life—EQ-5D-5L

The primary outcome measure is the average patient quality of life, over 6 months, assessed by the patient-reported outcome measure, EQ-5D-5L. EQ-5D-5L will be collected at baseline (for today and 1 week prior to injury (adapted with permission)), 2-week, 6-week, 12-week, and 6-month time points, as well as an optional 12-month follow-up point for those recruited early to the study and who reach this time point within the planned follow-up period.

The EQ-5D-5L is a validated generic patient-reported outcome measure (www.euroqol.org), including validation in patients with hip fractures and orthopaedic patients with cognitive impairment [26]. The descriptive system has five health domains (mobility, self-care, usual activities, pain/discomfort, and anxiety/depression) with five response options for each domain (no problems, slight problems, moderate problems, severe problems, and extreme problems). In addition, it has a health status visual analogue scale (VAS) which measures self-rated health with endpoints ranging from ‘the best health you can imagine’ to ‘the worst health you can imagine’. The EQ-5D-5L will be scored according to the user guide [27]. The measure is easily completed and can be completed by proxy (which is important for our clinical population), and it can also be scored for those who die during follow-up. EQ-5D-5L data will be collected in either patient questionnaires or proxy questionnaires for those who lack capacity. Details of how scores will be aggregated and analysed are given in the statistical methods section. The EQ-5D-5L will be also used to estimate quality-adjusted life years (QALYs) for the cost-effectiveness analysis.

#### Secondary outcomes

##### Physical function

Physical function will be measured using the Patient Reported Outcome Measures Information System (PROMIS) Lower Extremity Function and the Timed Up and Go test (TUG).

PROMIS Lower Extremity Function data will be collected in the patient questionnaires (or proxy questionnaires for those who lack capacity) at baseline and 2-week, 6-week, 12-week, and 6-month time points. PROMIS is a set of validated person-centred measures that evaluates physical, mental, and social health in adults and children [28]. The full item bank can be used for computer adaptive testing but is also available in a range of subscales and short forms to measure different aspects of health. Lower Extremity Function (Neuro-QOL Short Form v1.0 – Lower Extremity Function (Mobility)) is an extremely important outcome domain for people with an LC-1 fracture, due to the impact of the injury on the ability to mobilise. This brief measure (Lower Extremity Function), administered as a paper-based questionnaire, is designed to reduce respondent burden and has been deemed to have good face validity with our PPI group.

The TUG will be undertaken at a 12-week follow-up point only when the visit is conducted in the clinic setting (there will be no attempt to perform this where a remote visit is undertaken). This test assesses walking speed, mobility, balance, and fall risk. It is an established test used routinely in practice and has been validated for reliability [29, 30]. An LC-1 fracture can impact significantly on the ability to mobilise, and this clinic-based measure will complement the patient-reported outcome measure PROMIS Physical function.

##### Global mental health

Global mental health will be measured using the PROMIS Scale v1.2 – Global Health Mental 2a.

This is a two-question subscale on global mental health; it will be collected in the patient and proxy questionnaires at baseline and 2-week, 6-week, 12-week, and 6-month time points. The inclusion of this subscale was highly commended by our PPI group.

##### Pain

Pain will be measured using a visual analogue scale (VAS); this is a unidimensional measure of pain intensity in adults [31]. We will use a scale ranging from ‘no pain’ to the ‘worst imaginable pain’ to measure the average pain over the last week. This data will be collected from participants with capacity only, at baseline and 2-week, 6-week, 12-week, and 6-month time points as well as an optional 12-month follow-up point for those recruited early within the study.

##### Delirium

Delirium will be measured by the Abbreviated Mental Test Score (AMTS) and the 4AT Rapid Assessment Test for Delirium. These tests will be conducted at baseline, at 2 weeks, and at 12 weeks. The 12-week tests will indicate whether new-onset delirium is temporary or a permanent change.

AMTS is a short, verbal test widely used in clinical practice to screen for confusion and dementia [32, 33]. It is used across many areas of medicine, and despite being developed in 1972 [33], recent data confirms its validity in emergency admissions in older adults within UK hospitals [32].

*4AT Rapid Assessment Test for Delirium* is a short, practical instrument validated for detecting delirium, routinely used in clinical practice [34, 35]. The strengths of the 4AT Rapid Assessment Test for Delirium are that it can be used on patients that are drowsy or agitated (which is common after surgery), it does not require specialist training, and it takes less than 2 min to complete.

Post-operative delirium is a known complication for older individuals, particularly those with dementia. The incidence in a hip fracture surgery population has been calculated as 24% [36]. Therefore, its use as an outcome measure will be to monitor this potential adverse effect of surgery. Post-operative delirium is associated with higher costs, functional decline, increased length of stay, discharge to a nursing home or care home, and higher mortality [37]. Therefore, understanding which participants exhibit post-operative delirium will aid in the interpretation of the findings and outcomes post-intervention.

##### Imaging assessments

A radiologic assessment of the pelvis will be performed between 12 weeks and 6 months to assess the non-union or late displacement of the LC-1 fracture.

The rate of non-union or late displacement of LC-1 fractures that were treated initially non-operatively but which subsequently required internal fixation has been reported as 4% [3], although this figure is not well corroborated by other studies. Such patients typically have ongoing symptoms from their pelvis and signs of displacement or non-union would be evident on follow-up X-rays from 12 weeks onwards. It is therefore critical in this study to have X-rays of both surgically treated and non-operative control groups for comparison, regarding non-union or displacement of the pelvic ring.

##### Resource use

Information on resource use throughout patients’ hospital stays and at discharge will be collected to assess the impact on the NHS as part of the economic evaluation. Data collected in clinic case report forms (CRFs) will include length of hospital stay, medication, surgery details, and details of therapy during rehabilitation. The 2-week and late discharge CRFs will also collect details on any aids or adaptations required and any change of place of residence (e.g. own home to residential care home) relative to baseline. Resource use data will also be collected in the 12-week patient questionnaire, from patients with capacity only. This will include information on any re-admittance to the hospital, outpatient care received, and any additional medications, aids, or adaptions since discharge and return to work.

##### Complications and adverse events

Information on expected complications, including additional surgery, will be collected in the hospital CRFs at 2 weeks, at 12 weeks, and at discharge (if after 2 weeks). Expected complications that will be recorded will include (but not be limited to) the following: neurological complications, deep wound infection (using Centres for Disease Control (CDC) and prevention definition) [38], superficial infection (using CDC definition), rehospitalisation, re-operation (including removal of implant), and skin problems.

Lateral cutaneous nerve injury is an adverse event of special interest (AESI), and information on this will be collected on an adverse event (AE) form. Patients will also be asked about this in the 2-week, 6-week, 12-week, and 6-month questionnaires, as well as in the 12-month questionnaires for those who agree to this additional follow-up.

Information on any unexpected adverse events or any expected or unexpected adverse events that become serious adverse events (SAEs) will be reported on the appropriate AE or SAE report form as discussed below.

##### Mortality

Mortality rates of 10–15% have been reported in this population 6 months after the fracture. Therefore, checks will be made on patients’ status before mailing out follow-up questionnaires at 6 and 12 months. Mortality will be reported as an outcome at 6 months (and 12 months for those patients that agree to this additional follow-up) (Table 2).

**Table 2:**
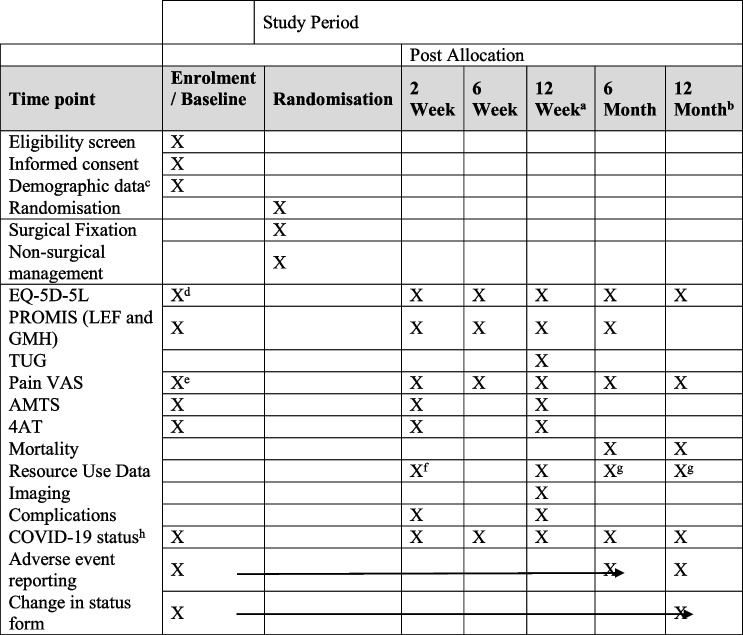
Participant timeline

^a^Visit may be conducted remotely in the event of local restrictions arising from COVID-19. TUG assessment will not be completed where the visit is remote. Radiology assessment may be performed up to the 6-month time point

^b^Optional follow-up time point for those patients that reach this time point within the planned follow-up period

^c^Patient demographic data collected will include the date of birth, gender, ethnicity, lifestyle, medical history and current medications, details of the fracture and any concomitant injuries, and Rockwood Frailty Score in the week prior to injury

^d^Data retrospectively collected for a week before the injury as well as on the day of baseline assessment

^e^This question will ask about their pain, since their injury only

^f^If the patient has not been discharged by the 2-week time point, health resource data will be collected via a review of medical records following the point of discharge

^g^Collected for patients with capacity only

^h^COVID-19 status will be recorded where routine testing has been undertaken on admission and patients will be asked to self-report the results of any additional testing undertaken during follow-up

### Sample size

It is estimated that 600 participants are required to address the study objectives. The primary outcome is the EQ-5D-5L over 6 months. To be conservative, we took the lowest published estimate of the minimal clinically important differences (MCID) (0.074) [39] with an estimated standard deviation of 0.25 (estimated from the 0.30 reported by Adachi et al. for the 3L version [40] and adjusted down to account for the 5L version’s greater sensitivity). Based on these assumptions, we would need to analyse 480 participants (240 per group), and after accounting for loss to follow-up of 20%, we would need to recruit and randomise 600 participants for a study with 90% power (2*p* = 0.05).

### Recruitment

The study will include an internal 12-month pilot phase to assess the assumptions about site set-up and recruitment.

We will prioritise the set-up of MTCs during the recruitment phase of the trial, training will be provided at site initiation visits, and training videos will be made available. Trial coordinators will provide ongoing guidance and support to the local principal investigators (PI), treating clinicians, and research staff at each centre to optimise screening and recruitment for their local circumstances. Clinical guidance will be sought from the CI as appropriate; email bulletins and email newsletters will be circulated to update the staff on trial progress and any relevant reminders.

Potentially eligible patients will be recruited from inpatient wards (surgical, elderly care, and medical). To identify eligible patients, the research associate will screen all patients 60 years old and over, admitted with an LC-1 fracture. Patient eligibility must be confirmed by a delegated surgeon or clinician. Eligible patients will be approached to discuss the study and given the patient information sheet, they will have time to discuss the study with their family and/or friends, and they will have the opportunity to ask questions of the surgeon and the local research team.

The research team will share ideas of best practice from other sites, and we will seek advice from PPI members to develop strategies to maximise patient recruitment.

#### Internal pilot

An internal 12-month pilot will address the question of whether there are a sufficient number of eligible patients identified and recruited in 12 months to make the trial viable within the proposed 36-month recruitment period.

The progression criteria agreed upon with the funder to be assessed at the end of the pilot will be to have a minimum of 19 sites open to recruitment, to achieve a recruitment rate of 1 patient/per month/per site (total of 148 patients randomised).

An average recruitment rate of one patient per centre per month would support a decision to progress to the main trial. An average rate of 0.80 to 0.99 per centre per month would suggest that a decision to progress may be supportable depending on other supplementary information available (e.g. number and characteristics of potential participants not approached, proportion not meeting eligibility criteria and reasons, proportion declining participation and reasons why) and whether any of the factors impeding recruitment could be remedied.

### Allocation of interventions

The online L1FE Data Management System is an independent secure randomisation service for sequence allocation hosted by York Trials Unit (YTU) and accessed by the research staff either by telephone or via the Internet. The research staff who have been delegated the responsibility to randomise patients on their site delegation log will be granted access using a personal login.

Once an eligible patient has consented and their baseline forms have been completed, the research staff will record their information on the L1FE Data Management System. The system will confirm eligibility and then perform independent and concealed random allocation (1:1), using computer-generated random permuted blocks of random sizes, stratified by centre. The patient will be allocated to either surgical fixation or non-surgical management.

Patients and treating clinicians will be informed of the allocation. As with many surgical trials, where the surgical site is clearly visible, it is not feasible to blind patients, surgeons, or outcome assessors. The primary outcome is a patient-reported measure, mitigating surgeon influence. All staff involved in analysing questionnaire responses will be blind to the patients’ treatment allocation.

### Data collection and management

Paper CRFs will be used to collect and record all outcome data. Data will be collected at recruiting sites by research staff on hospital CRFs and participants will complete CRFs by post. All CRFs will be returned to YTU for scanning and processing. All reporting of data collection will be undertaken in line with the Consolidated Standards of Reporting Trials (CONSORT) statement [41].

To minimise attrition, we will use multiple methods to keep in contact with participants. We will ask participants for full contact details (including mobile phone numbers and email addresses). We will also collect alternative contact details of someone who can be contacted if the participant changes address. Participants can complete the 2- and 12-week questionnaires in the clinic when attending in person or they can be completed over the phone. The 6-week, 6-month, and 12-month questionnaires will be either completed by post or over the phone for the patient’s convenience. Pre-notification letters will be sent out before the postal follow-up questionnaires are due, to help prime participants, and a text message reminder will also be sent on the day participants are expected to receive the postal questionnaire. This has been shown to significantly reduce the time to questionnaire response [42]. There will also be 2 follow-up postal reminders and a telephone reminder at each time point if required. The telephone reminder will give participants the option to complete an abridged questionnaire (a minimum of the EQ-5D-5L). The study team will also call the participant when there is missing data on the primary outcome (and other missing data as feasible) when a postal questionnaire is returned. We will also write newsletters during the trial to keep the participants informed and engaged with the trial which can enhance response rates [43].

Participants are free to fully withdraw from the study at any point; however, it is also possible for them to withdraw from only one aspect of the trial if participation becomes a burden. For example, participants can continue with either clinical visits only, postal questionnaires only, or data collection from their hospital records only with no participant involvement. It is anticipated that these options will reduce the need for patients to fully withdraw from the trial and enable some useful data to still be collected.

Improving the retention of participants is important to all RCTs, and there is a need to develop and test interventions to improve retention. The L1FE trial will act as a host trial for an embedded trial, referred to as a Study Within A Trial (SWAT). The objective of this SWAT is to evaluate the impact of making a courtesy introductory telephone call to newly recruited trial participants on response rates to follow-up questionnaires compared with a written card with equivalent information, or nothing. This SWAT is registered on the MRC SWAT Repository. SWAT Ref 114: Effects of telephone calls or postcards to trial participants following enrolment on retention in a randomised trial.

### Data management

An electronic management system will be used to track participant recruitment and study status as well as CRF returns. Data from CRFs will be processed by administrative personnel at YTU. Data will be verified through cross-checking of the data against the hard copy of the CRF. The trial coordinator and statistician will write a validation plan for the CRFs in consultation with the YTU Data Manager. The plan will include detailed coding for the CRFs and data query resolution rules/procedures. Quality control will be applied at each stage of data handling to ensure that all data are reliable and have been processed correctly.

All paper records will be kept in locked locations for the duration of the study.

### Confidentiality

Data will be handled in accordance with the Data Protection Act 2018, General Data Protection Regulation (GDPR) legislation, the latest Directive on Good Clinical Practice, and local policy.

All personal information collected about enrolled participants will be held electronically in a secure environment at the University of York, with permissions for access in line with standard operating procedures (SOPs). All paper records containing personal information such as consent forms and consultee declaration forms will be stored safely in a separate compartment of a locked cabinet.

Clinical information will only be looked at by responsible individuals from the study team, the sponsor, the NHS Trust, or regulatory authorities, where it is relevant to the patient taking part in this research as he/she would have agreed to at the time of consent or consultee declaration.

The researchers and clinical care teams must assure that patients’ anonymity will be maintained and that their identities are protected from unauthorised parties. Once randomised, patients will be assigned a participant ID number. This will be used on all CRFs, and individual participants will only be referred to by their participant ID number to maintain patient confidentiality. All study data will be completely anonymised for any analyses, reports, or publications.

## Statistical methods

### Statistical methods for primary and secondary outcomes

Full analyses will be detailed in a statistical analysis plan (SAP), which will be finalised prior to the end of data collection and which will be reviewed and approved by the independent data monitoring committee. Any exploratory analyses of sub-groups that are of clinical interest will be pre-specified in the SAP. This trial will be reported according to the CONSORT guidelines for clinical trials.

Statistical analyses will be on an intention-to-treat basis with patients being analysed in the groups to which they were randomised. Analyses will be conducted using 2-sided significance tests at the 5% significance level (unless otherwise stated in the SAP).

A CONSORT flow diagram will be provided to display the flow of participants through the study. The number of participants withdrawing from the trial will be summarised with reasons where available. Baseline characteristics will be presented by trial arm. All trial outcomes will be reported descriptively by trial arm at all time points at which they were collected. Continuous baseline and outcome data will be summarised as means, standard deviations, medians, and ranges, whereas categorical data will be summarised as frequencies and percentages.

The primary analysis will be a mixed effects linear regression model, with EQ-5D-5L scores at 2-week, 6-week, 12-week, and 6-month time points as the dependent variable, adjusting for baseline EQ-5D-5L, randomised group, and other pertinent baseline characteristics as fixed effects. Potential clustering at the hospital site level will be controlled by including it in the model as a random effect. The model will account for the correlation of scores within patients over time by means of an appropriate covariance structure. The estimated treatment group differences across all time points will be reported as the primary endpoint with 95% confidence interval and associated *p*-value. Secondary analyses will include an estimate of treatment group differences at each time point from the same model.

The secondary outcomes: PROMIS: Lower Extremity Function score, TUG score, and AMTS score, 4AT score; PROMIS Scale v1.2: Global Health Mental 2a score and Pain VAS, will be analysed by similar mixed effects linear regression models. Mortality will be analysed using a logistic regression model.

An economic evaluation analysis will be conducted from the recommended NHS and personal social services (PSS) perspective according to National Institute for Health and Clinical Excellence (NICE) guidance [18]. Data will be collected on the costs and outcomes of each trial participant during the period between randomisation and 6 months post-randomisation as well as an optional 12-month time point. The internal pilot phase will permit testing of the data collection forms to be used in the economic analyses in terms of validity, consistency, reliability, and response rate (e.g. missing data). Trial participants will be asked to complete economic resource use questionnaires at 12 weeks and 6 months as well as at the optional 12-month time point. These will report hospital (e.g. inpatient, outpatient, A&E), community, and social care resources used, and for the purposes of secondary analysis, costs associated with lost productivity and out-of-pocket costs. Hospital forms will be specifically designed to collect information on the cost of surgery (e.g. time in theatre, staff time, consumables and devices, nights in hospital after the procedure), complications, physiotherapy, and removal of devices. Relevant UK unit costs, such as NHS Reference costs and PSS Research Unit costs of health and social care, will be applied to each resource item to value total resource use in each group.

Health outcomes will be expressed in terms of the quality-adjusted life years (QALYs) using the EQ-5D-5L data collected at baseline, 2 weeks, 6 weeks, 12 weeks, and 6 months post-randomisation. The EQ-5D-5L health states will be valued following the NICE position statement [44]. QALYs will be calculated using the area under the curve analysis [45].

Costs and QALYs will be synthesised to generate an incremental cost-effectiveness ratio (ICER). Regression methods will be used to allow for differences in prognosis variables. The pattern of missing data will be analysed and handled by means of multiple imputation (MI) methods if deemed appropriate according to the missing data pattern in the L1FE dataset [46]. A range of sensitivity analyses will be conducted to test the robustness of the results using different scenarios. The uncertainty will be presented using cost-effectiveness acceptability curves. The probability that each intervention is cost-effective will be reported at the cost-effectiveness thresholds of £20,000 to £30,000/QALY and £13,000/QALY as suggested by recent research [47, 48], if results deem appropriate (i.e. there is a non-dominant situation in the trial-based evaluation). We will undertake a secondary analysis to extrapolate the results of the trial beyond the study follow-up.

A detailed economic analysis plan will be agreed upon with the TSC before all data has been collected.

### Interim analyses

There is an internal 12-month pilot phase to assess the feasibility and recruitment rate of this study.

Detailed screening logs will be kept by participating centres and the recruitment rate will be reported by month, by hospital site and overall from the data collected. A CONSORT diagram will be constructed to show the flow of participants through the study and the following outcomes calculated: number of eligible patients, proportion of eligible patients approached for consent, proportion of eligible patients not approached and reasons why, proportion of patients approached who provide consent, proportion of patients approached who do not provide consent and reasons why, proportion of patients providing consent who are randomised, proportion of patients randomised who do not receive the randomly allocated treatment and reasons why, and proportion of patients dropping out between randomisation and follow-up and reasons why. For each of the above, we will collect data on whether consent was sought from a patient or for patients who lack the capacity whether a personal or nominated consultee was asked to give a declaration.

Details of all participating surgeons’ prior experience with the INFIX procedure will be collected as part of the trial. Equipoise is an essential concept in trials and will be covered during the training delivered as part of the site setup process. The assumption of surgeon equipoise will be monitored during recruitment by scanning reasons for exclusion during screening and reasons for crossover following randomisation that may reflect surgeon preferences.

These data will be compared against the study’s recruitment assumptions and progression targets to inform the continuation of the trial or relevant modifications to improve recruitment rates. The final decision on the progression from the pilot phase to the main trial will be made by the funding body.

### Methods for additional analyses (e.g. subgroup analyses)

A subgroup analysis will be performed to explore the potential effect of patients’ knowledge of which treatment they received (allocation cannot be blinded) and their experience of this treatment on the results of the trial. This will be for the primary outcome only, and the interaction term between preference and treatment group will be included in the primary analysis model as described in the previous section.

For patients who are eligible and opt-in to complete the 12-month follow-up questionnaire, the primary analysis model will be extended and the treatment effect with the associated 95% CI reported for the 12-month follow-up time point.

### Methods in analysis to handle protocol non-adherence and any statistical methods to handle missing data

The number of participants not receiving their allocated treatment will be reported by group. In the presence of non-adherence with randomised treatment, a CACE analysis will be undertaken using an instrumental variable regression model.

The primary analysis model will use a mixed effects regression model which implicitly assumes missing outcome data are missing at random (MAR). However, it is possible that participants who failed to complete their follow-ups differed from those who did complete them. This would mean the data were missing not at random and would represent a departure from the MAR assumption. The sensitivity of the primary analysis results to departures from the MAR assumption will be explored using a pattern-mixture model, implemented using the rctmiss command.

### Plans to give access to the full protocol, participant-level data, and statistical code

The full protocol is available via the funder website: https://www.fundingawards.nihr.ac.uk/award/16/167/57. Requests for other data or documentation should be made by contacting the corresponding author.

## Oversight and monitoring

The primary responsibility for monitoring the safety of participants in clinical trials lies with the trial sponsor. Data monitoring will be undertaken by the Trial Management Group (TMG), Trial Steering Committee (TSC), and a Data Monitoring and Ethics Committee (DMEC), on behalf of the sponsor and funder. The project will also be monitored by the sponsor for whom a representative will be invited to attend the TMG and TSC meetings. Regular progress reports will be submitted to the funding body.

The TMG will oversee the day-to-day management of L1FE and is chaired by the CI. Other members include the trial statisticians, trial manager, trial coordinators, health economist, and other co-applicants. The role of the TMG is to monitor all aspects of the conduct and progress of the trial, ensure that the protocol is adhered to, and take appropriate action to safeguard participants and the quality of the trial itself. The TMG will meet monthly by video or teleconference from the start of the study until the end of the pilot phase and quarterly for the remainder of the study.

The TSC is independent and has been established to provide overall supervision for L1FE on behalf of the sponsor and project funder and to ensure that the project is conducted to the rigorous standards set out in the Department of Health’s Research Governance Framework for Health and Social Care and the Guidelines for Good Clinical Practice (GCP). This committee comprises an independent chair who is a professor of clinical trials, a consultant orthopaedic surgeon with expertise in the procedure, a public contributor, a consultant physiotherapist, a representative from the sponsor, the CI, and the trial coordinator/manager. Other study collaborators may also attend the meeting with the agreement of the chair. The TSC will meet at least annually and will work to an agreed charter.

The DMEC is chaired by a statistician, with other members comprising experts in the clinical area: professor of trauma and orthopaedics, senior lecturer in physiotherapy, and the CI. The role of the DMEC is to review accumulating data in L1FE and advise the sponsor (directly or indirectly) on the future management of the trial. The DMEC will review safety and efficacy data as well as quality and compliance data. The DMEC will review all serious adverse events which are thought to be treatment related and unexpected. The independent members of the DMEC will be allowed to see unblinded data. The DMEC will meet at least annually or more frequently if the committee requests, and will work to an agreed charter.

Any substantial amendments will be submitted to the Health Research Authority (HRA) (and Research Ethics Committee (REC) where required) having been agreed with the funding body, sponsor, TSC, DMEC, and the TMG. Minor modifications to the protocol will be agreed with the TMG and sponsor before submission for approval to the HRA. All amendments will be implemented in the NHS organisations in agreement with the guidance and approval of the HRA. All amendments will be listed in the published final report to the funding body.

### Adverse event reporting and harms

Due to the age of this patient population and likelihood of unrelated AEs occurring, any expected AEs will be considered complications, and data will be collected on these in the CRFs as described above.

We will collect data for the AESI and any unexpected adverse events that are related to treatment for the original injury. We will collect AE data from the point of randomisation to 6 months post-randomisation for all patients and up to 12 months post-randomisation for patients that agree to this additional time point. All AEs will be listed on the appropriate AE CRF for routine return to YTU.

Expected and unexpected SAEs will be reported if they appear to be related to any aspect of taking part in the study and occur within 6 months of randomisation for all patients and up to 12 months post-randomisation for patients that agree to this additional time-point. All SAEs will be entered onto the SAE reporting form and forwarded to YTU within 24 h of the investigator becoming aware of them. Once received, causality and expectedness will be confirmed by the CI. SAEs that are deemed to be unexpected and related to the trial will be notified to the Research Ethics Committee (REC) and sponsor within 15 days. All such events will be reported to the TSC and DMEC at their next meetings. Follow-up reports a month later may be requested by the CI for their review to ensure that adequate action has been taken and progress made. All participants experiencing SAEs will be followed up as per protocol until the end of the trial.

The summary of complications, deaths, AEs, and SAEs experienced by the participants will be reported by treatment group.

### Frequency and plans for auditing trial conduct

Central monitoring will be undertaken, with triggered on-site monitoring if significant issues are identified. Direct access will be granted to authorised representatives from the sponsor, host institution, and the regulatory authorities to permit study-related monitoring, audits, and inspections.

### Dissemination plans

Through the planned outputs, the study is expected to play a key role in enhancing the evidence base on the effectiveness and cost-effectiveness of surgical fixation for the management of pelvic fractures. The economic component will help us to identify the most efficient provision of future care and thus savings to the NHS and society.

The executive summary and copy of the trial report will be sent to NICE and other relevant bodies, including Clinical Commissioning Groups, so that study findings can inform their deliberations and be translated into clinical practice nationally. We will work with the relevant Specialty Advisory Committees (SAC) to incorporate the findings into the training curriculum for clinicians who will undertake treatment for pelvic fractures. We will use several dissemination channels to ensure that patients and the public are also informed about the results of the study. We will produce the following outputs:

- A HTA research monograph will be produced.
- In conjunction with patient members of the team, we will generate patient information for ‘shared decision-making’ based on the findings from this trial.
- The results of the study will be presented at national and international surgical meetings such as the British Orthopaedic Association Annual Congress, the UK Orthopaedic Trauma Society meeting, the North American Orthopaedic Trauma Association, the European Federation of National Associations of Orthopaedics and Traumatology (EFFORT), Société Internationale de Chirurgie Orthopédique et de Traumatologie (SICOT), and the American Academy of Orthopaedic Surgeons.
- The results will be shared with relevant charities such as the Royal Osteoporosis Society to ensure wider dissemination amongst the public. The results will also be presented at meetings led by the Chartered Society of Physiotherapy and the Royal College of Physicians to teach the wider multidisciplinary team.
- The findings will be published in peer-reviewed high-impact general medical and orthopaedic journals such as *Lancet*, the *BMJ*, or similar.
- A summary of the study report, written in lay language, will be produced and made available to participants, members of our user group, and relevant patient-focused websites.
- We will seek to raise the profile of the trial via social media including a dedicated Twitter account. This will be aimed at participating site staff and focus on trial progress, trial-related events, and publicising research outputs.
- If found to be effective, the MTC pelvic specialist surgeon co-applicants will explore ways of cascading training in the technique to orthopaedic surgeons in NHS hospital trauma units to ensure consistency of best practice across the NHS.

## Discussion

This study aims to further the knowledge of treatment options for patients aged 60 years and older with LC-1 fragility fractures, a common and painful injury that can lead to long-term immobility in some patients. The clinical and cost-effectiveness of two treatment options will be compared; these are non-surgical management, the current standard of care and INFIX surgery, an internal fixation device which is frequently used to stabilise this fracture in younger patients. The study has an inbuilt 12-month pilot phase to test the feasibility of recruiting to this study. The results will be disseminated through peer-reviewed publications, and the evidence will help to inform clinical practice.

## Trial status

The current version of the protocol is L1FE Trial Protocol V3.2, 22nd December 2020.

Recruitment to the L1FE trial started in August 2019, and recruitment was anticipated to be completed at the end of March 2022. However, recruitment to the L1FE study was suspended in March 2020 due to the COVID-19 pandemic. Amendments were made to the trial protocol in response to the pandemic, to increase the flexibility of follow-ups with the option for more data collection to be done remotely. Recruitment restarted on 15 March 2021 with a plan to continue the initial 12-month internal pilot phase for a further 6 months until 15 September 2021. On 13 August 2021, the decision was made that the study was not feasible, in part due to a change in patient pathways in response to the COVID-19 pandemic. All sites were notified to stop recruitment with immediate effect.

At the time of manuscript submission, enrolled patients remain in the follow-up phase with the last patient last visit anticipated in November 2021.

## Supplementary Information

**Additional file 1. **SPIRIT 2013 Checklist: Recommended items to address in a clinical trial protocol and related documents*. *It is strongly recommended that this checklist be read in conjunction with the SPIRIT 2013 Explanation & Elaboration for important clarification on the items. Amendments to the protocol should be tracked and dated. The SPIRIT checklist is copyrighted by the SPIRIT Group under the Creative Commons ‘Attribution-NonCommercial-NoDerivs 3.0 Unported’ licence.

## Data Availability

Permission to access source data by the study staff and for regulatory and audit purposes will be sought via the patient consent form, with an explicit explanation in the information sheet and consent discussion. External requests for data following the completion of planned analysis and dissemination will be notified to the CI and sponsor for consideration and approval before seeking confirmation from the funding body. Any data will be anonymised before secure transfer.

## Abbreviations

AE: Adverse event
AESI: Adverse event of special interest
CEAC: Cost-effectiveness acceptability curves
CI: Chief investigator
CONSORT: Consolidated Standards of Reporting Trials
CRF: Case report form
DMEC: Data Monitoring and Ethics Committee
EQ-5D-5L: EuroQol 5 Dimension, 5-Level Scale
GCP: Good Clinical Practice
GDPR: General Data Protection Regulation
HES: Hospital Episode Statistics
HRA: Health Research Authority
ICER: Incremental cost-effectiveness ratio
LC-1: Lateral compression type 1
MTCs: Major trauma centres
NHS: National Health Service
NICE: National Institute for Health and Clinical Excellence
NRS: Numerical Rating Scale
ONS: Office of National Statistics
PI: Principal investigator
PPI: Patient and Public Involvement
PROMIS: Patient Reported Outcome Measures Information System
PSS: Personal social services
QALY: Quality-adjusted life year
RCT: Randomised controlled trial
REC: Research Ethics Committee
SAE: Serious adverse event
SAP: Statistical analysis plan
SD: Standard deviation
SDV: Source data verification
SOP: Standard operating procedure
SWAT: Study Within A Trial
TMF: Trial Master File
TULIP: Trial of surgical versus non-surgical treatment of lateral compression injuries of the pelvis with complete sacral fractures (LC-1) in the non-fragility fracture patient—a feasibility study
TUG: Timed Up and Go Test
VTE: Venous thromboembolism
YTU: York Trials Unit
